# Predicting the number of article citations in the field of attention-deficit/hyperactivity disorder (ADHD) with the 100 top-cited articles since 2014: a bibliometric analysis

**DOI:** 10.1186/s12991-021-00329-3

**Published:** 2021-01-21

**Authors:** Chien-Ho Lin, Tsair-Wei Chien, Yu-Hua Yan

**Affiliations:** 1 Department of Psychiatry, Chi Mei Medical Center, Tainan, Taiwan; 2 Department of Medical Research, Chi-Mei Medical Center, No. 901, Chung Hwa Road, Yung Kung Dist., Tainan, 710 Taiwan; 3 Department of Medical Research, Tainan Municipal Hospital (Managed By Show Chwan Medical Care Corporation), No. 670, Chung Te Road, Tainan, 701 Taiwan; 4 Department of Hospital and Health Care Administration, Chia Nan University of Pharmacy and Science, No. 1, Changda Rd., Gueiren District, Tainan, 71101 Taiwan

**Keywords:** Bibliometric, Citation analysis, Social network analysis, Medical subject heading, Attention-deficit/hyperactivity disorder, Correlation coefficient

## Abstract

**Background:**

Attention-deficit/hyperactivity disorder (ADHD) is a common neurodevelopmental disorder in children or early adolescents with an estimated worldwide prevalence of 7.2%. Numerous articles related to ADHD have been published in the literature. However, which articles had ultimate influence is still unknown, and what factors affect the number of article citations remains unclear as well. This bibliometric analysis (1) visualizes the prominent entities with 1 picture using the top 100 most-cited articles, and (2) investigates whether medical subject headings (i.e., MeSH terms) can be used in predicting article citations.

**Methods:**

By searching the PubMed Central^®^ (PMC) database, the top 100 most-cited abstracts relevant to ADHD since 2014 were downloaded. Citation rank analysis was performed to compare the dominant roles of article types and topic categories using the pyramid plot. Social network analysis (SNA) was performed to highlight prominent entities for providing a quick look at the study result. The authors examined the MeSH prediction effect on article citations using its correlation coefficients (CC).

**Results:**

The most frequent article types and topic categories were research support by institutes (56%) and epidemiology (28%). The most productive countries were the United States (42%), followed by the United Kingdom (13%), Germany (9%), and the Netherlands (9%). Most articles were published in the Journal of the American Academy of Child and Adolescent Psychiatry (15%) and JAMA Psychiatry (9%). MeSH terms were evident in prediction power on the number of article citations (correlation coefficient = 0.39; *t* = 4.1; *n* = 94; 6 articles were excluded because they do not have MeSH terms).

**Conclusions:**

The breakthrough was made by developing 1 dashboard to display 100 top-cited articles on ADHD. MeSH terms can be used in predicting article citations on ADHD. These visualizations of the top 100 most-cited articles could be applied to future academic pursuits and other academic disciplines.

## Background

Attention-deficit/hyperactivity disorder (ADHD) is a common neurodevelopmental disorder in children or early adolescents [1] with an estimated worldwide prevalence of 7.2% [2]. In 60% of affected individuals, ADHD symptoms persist until adulthood [3]. Consequently, ADHD occurs more frequently in males than in females in a ratio of 3–5:1 [4, 5].

The view of ADHD as a multifactorial disorder with a genetic component comes from the clinical complexity observed in its symptomatology. Changes occur throughout the life span, with younger children displaying more hyperactive–impulsive behaviors and adolescents and adults exhibiting more symptoms of inattention [6]. Over the last 2 decades, there have been numerous technical and methodological advances available to clinicians and researchers to better understand ADHD and its etiology. Despite the growing body of literature investigating the pathophysiology of ADHD, it remains a complex psychiatric disorder difficult to characterize [7].

Bibliometric methods have been widely used to analyze books and articles and assess the impact of research outputs [8]. This type of analysis identifies the countries, organizations, and the authors who had the most prominent scientific contributions [9, 10]. The topics, study design, and levels of evidence-based medicine (EBM) of highly cited articles may influence the trends in clinical practice and further research [11–13]. The number of citations of an article usually indicates the interest of the researchers on using the articles referred to their own studies. Accordingly, bibliometric analysis can be used to summarize the status quo and development trends of a specific disease or research field, providing ideas and directions for future research [14, 15].

Many medical specialists have utilized citation rank analysis to identify the most influential papers in their field, which include drug [16], disease [17, 18], cancer [19], and surgery [20, 21]. To date, no studies have been undertaken to determine the most influential papers in the field of ADHD. Through this study, the authors analyze 100 top-cited articles on ADHD through a systematic search strategy using two required approaches: (1) visualization of prominent entities with one picture and (2) investigation whether medical subject headings (i.e., MeSH terms defined in PubMed Central^®^) can be used in predicting article citations.

## Methods

### Data source

Two steps were conducted for data organization. First, the authors searched the PubMed Central^®^ (PMC) using the keywords ((Attention-Deficit/Hyperactivity Disorder (ADHD))) AND (("2014"[Date—Publication]:"3000"[Date—Publication])) as of May 29, 2020 and downloaded 13,629 abstracts since 2014.

Second, based on the article types and topic categories with MeSH terms shown in PMC, the authors extracted the top 100 articles ranked by the number of citations. (1) The main contributors from countries/journals and (2) the prediction power of article citations related to MeSH terms were presented using figures and tables. Since all data were obtained from a publicly available database, this study does not require ethical approval.

### Data arrangement

Based on the titles and abstracts of the top 100 most-cited articles on ADHD, 10 article types were identified by the PMC library and 8 topic categories with MeSH terms were clustered using social network analysis (SNA) [22].

The contributors to ADHD were examined using the top 100 most-cited articles shown on a dashboard. The authors tabulated document counts over the years with impact factors (IF) for countries of origin and the cross-relationship between article types and journals.

### Visualization and dashboard

Social network analysis [22] was performed to cluster entities (including countries of origin, journals, and MeSH terms) related to the article types and topic categories. The closer entities (e.g., MeSH terms) will appear in an identical subnetwork (or say cluster). Then, relevant entities will be highlighted in the subnetwork. This dashboard displaying the article types and relevant entities was laid on Google Maps.

### Prediction effect on article citations

The IFs of MeSH terms were computed based on the proportions and citations in an article. The weighted scores yielded by MeSH weights (i.e., the number of citations per article) in each article were used to predict original citations.

### Statistics

The correlation coefficient (CC) is used to determine the prediction power between the weighted MeSH terms and original article citations. The CC t-value is calculated by the following formula:$= \left(\frac{CC}{\sqrt{\frac{1-{\mathrm{CC}}^{2}}{n-2}}}\right)$. A prediction equation was produced using the simple regression analysis using the MedCalc statistical software, version 9.5.0.0 (MedCalc, New York, New York, USA). The significance level was set at Type I error (0.05). The study process is presented in Additional file 1: Video S1.

## Results

**Fig. 1:**
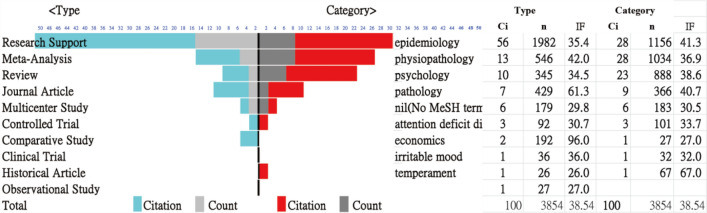
Citation rank analysis of article types and topic categories

The results of the top 100 most-cited articles on ADHD were elucidated at the reference [23], where readers were invited to examine all 100 articles included in this study. The citation counts for the 100 articles ranged from 22 to 220 as of May 28, 2020, in PMC, with an average of 38.54 citations. The most frequent article types and categories were research support (i.e., defined by PMC library and deemed as the study was supported by grants or funds form institutes or the government) by researcher’s institutes (56%) and epidemiology (28%) (Fig. 1).

The most productive countries were the United States (39%), followed by the United Kingdom (13%), Germany (9%), and the Netherlands (9%) (Table 1). Most articles were published in the Journal of the American Academy of Child and Adolescent Psychiatry (15%) and JAMA Psychiatry (9%) (Table 2).

**Table 1:** The trend of publications on ADHD between 2014 and 2018

Country	2014	2015	2016	2017	2018	2019	*n*	Ci	IF
US	22	6	8	2	1			39	1712
UK	8	1	2	2				13	433
Germany	7	2						9	325
Netherlands	4	4	1					9	278
Sweden	5		1					6	204
Australia	3	1	1					5	259
Denmark	1	3				1		5	192
Canada		1	1					2	59
China	2							2	53
Italy	1		1					2	71
Spain	2							2	76
Brazil			1					1	33
Bulgaria	1							1	24
Finland			1					1	34
Israel	1							1	39
Norway				1				1	25
Switzerland	1							1	37
N	58	18	17	5	1	1		100	3854

**Table 2:** The top 100 most-cited journals on ADHD between 2014 and 2018

Journal	A1	A2	A3	A4	A5	A6	A7	A8	A9	A10	n	CI	IF
J Am Acad Child Adolesc Psychiatry					1	7			7		15	677	45.1
JAMA Psychiatry		1					1		7		9	357	39.7
Biol Psychiatry						1			6		7	266	38.0
J Child Psychol Psychiatry			1			1		1	4		7	227	32.4
Pediatrics		1					1		3		5	259	51.8
Eur Child Adolesc Psychiatry									2	1	3	94	31.3
Hum Brain Mapp							1		2		3	105	35.0
J Abnorm Child Psychol					1				1	1	3	103	34.3
Atten Defic Hyperact Disord					1				1		2	59	29.5
BMJ							1		1		2	62	31.0
Environ Health Perspect						1			1		2	56	28.0
J Abnorm Psychol					1				1		2	59	29.5
J Neurosci									2		2	63	31.5
JAMA Pediatr									2		2	85	42.5
Mol Psychiatry									2		2	53	26.5
Neuropsychiatr Dis Treat										2	2	71	35.5
Neuropsychopharmacology			1							1	2	94	47.0
PLoS One									2		2	55	27.5
Addict Behav									1		1	31	31.0
Annu Rev Clin Psychol									1		1	41	41.0
Others	1	0	1	1	3	3	2	0	10	5	26	1037	39.9
Total	1	2	3	1	7	13	6	1	56	10	100	3854	38.54

A1, clinical trial; A2, comparative study; A3, controlled trial; A4, historical Article; A5, journal article; A6, meta-analysis; A7, multicenter Study; A8, observational study; A9, research support; A10, review

**Fig. 2:**
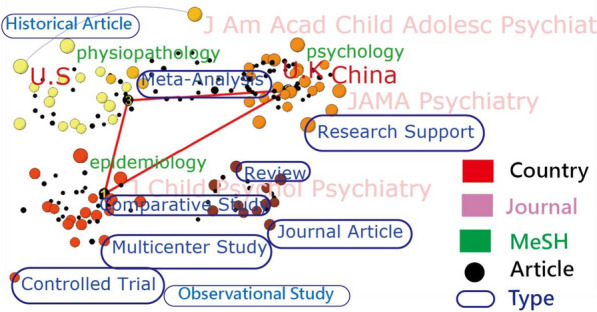
Top two entities among different variables

The most-cited article (PMID = 24,342,384) with 276 citations [24] was authored by Dr. Visser from the Developmental Disabilities group of the Centers for Disease Control and Prevention (CDC) in the United States. All the most dominant entities were highlighted on a visual board (Fig. 2). Readers are invited to click on the link at the reference [25] and examine the details or other relevant entities on the dashboard.

It is worth noting that the 10 article types are surrounded by other entities in Fig. 2 [25]. Through viewing the text colors, it is easy to discriminate the countries (red), MeSH terms (green), and article types (blue), which is different from traditional word clouds [26], only displaying one entity(or attribute) in a picture. The top three articles are linked by the triangle lines at the top-left corner in Fig. 2. Readers are invited to click on the black bubble of interest [25]. The abstract immediately appears for reading on PMC.

**Fig. 3:**
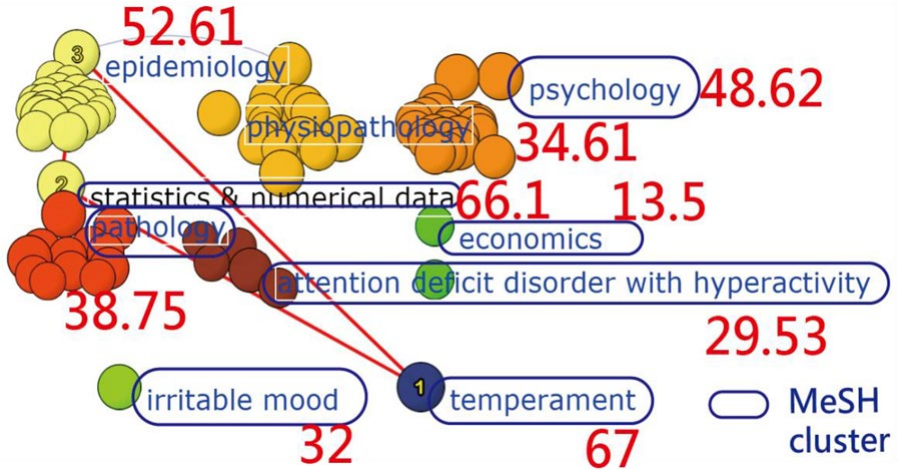
MeSH clusters and MeSH weights

For citation rank analysis, eight topic categories with MeSH terms are clustered in Fig. 3. The top three topics with the largest numbers of weights for predicting article citations are temperament (67), statistics and numerical data (66.1), and epidemiology (52.61), shown in Fig. 3 [27].

**Fig. 4:**
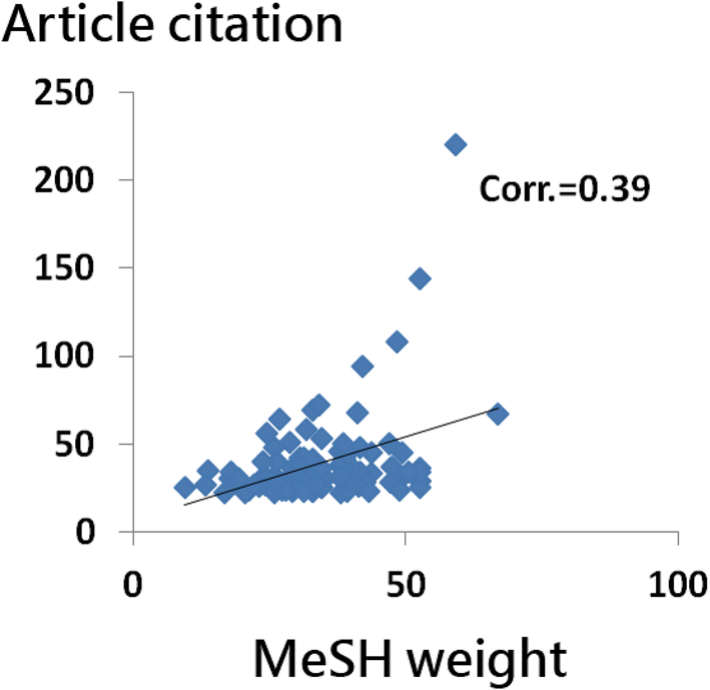
Use of MeSH weights in predicting article citations

Using the MeSH weights to predict article citations based on the authors’ research result, MeSH terms were useful in predicting the number of article citations (CC = 0.39, *t* = 4.1; *n* = 94; 6 articles were excluded because they do not have MeSH terms) (Fig. 4). The regression equation is defined as article citation (*y*) = 6.1217 + 0.9552 × weight (*x*) of MeSH terms. The slope coefficient showed statistical significance (F = 16.82; *p* < 0.001).

## Discussions

The authors conducted a bibliometric analysis of the top 100 most-cited articles on ADHD since 2014 using the publications from PMC. Open biomedical research on articles from the United States National Library of Medicine (i.e., the creator of PMC) on an international scale is gaining traction. This built an open biomedical and life sciences repository of freely accessible full-text journal literature in PMC in 2000 [28].

### Dominant countries and journals on ADHD

The authors identified and characterized the top 100 most-cited articles on ADHD. It may enable the identification of trends in ADHD studies and provide a historical perspective on scientific progress in this field. The authors evaluated the 100 most influential articles related to ADHD in history. In this study, nearly half of the papers came from the United States (*n* = 39) and the United Kingdom (*n* = 13). This is consistent with other bibliometric studies where the United States contributed most of the publications.

The United States is a dominant country in terms of contributions to the development of ADHD, having the largest numbers of most-cited articles, scientists, and research institutions. The United States has a strong influence on research in the health sciences; this can be attributed to the high level of financial grant support given to research in that country and a large number of American researchers [29]. Indeed, the United States is the leading country regarding medical research publications. Furthermore, the United Kingdom ranked second. Based on a recent surveillance of the need for transition in the United Kingdom, a very conservative estimate of the annual incidence of young people with an ongoing need of medications for ADHD lies between 270 and 511 per 100,000 people aged 17–19 years. Therefore, in the United Kingdom, there are more patients with ADHD [30]. In addition, the Netherlands ranked third; this roughly correlates with 95,000 older adults who have syndromic ADHD and 145,000 older adults who have symptomatic ADHD [31].

Over 46 journals were involved in this study, with the Journal of the American Academy of Child and Adolescent Psychiatry (15%) being the most frequent one, followed by JAMA Psychiatry (9%) and Biological Psychiatry (7%), indicating that the researchers followed these three journals, which are publishing new information regarding ADHD frequently. These journals are generally the top ones in the medicine field. In addition, recently published papers may not have sufficient citations mainly because citation rank analysis is time dependent [31].

### Three most-cited articles

The top-ranked article was titled “Trends in the Parent-Report of Health Care Provider-Diagnosed and Medicated Attention-Deficit/Hyperactivity Disorder: United States, 2003–2011” authored by Visser et al., in 2011 [24]. It was cited 220 times. In the years between 2003 and 2011, about 2 million more children/adolescents in the United States were diagnosed with ADHD. It draws public concern on the mental health of children and adolescents. As the need for accurate diagnosis and effective management of ADHD increases, this article was thus frequently cited in many articles.

The second-ranked article was authored by Thompson et al. and titled “The ENIGMA Consortium: Large-Scale Collaborative Analyses of Neuroimaging and Genetic Data,” which was published in 2014 [32]. It was cited 199 times. The ENIGMA Consortium was a huge study that included a collaborative network across 70 institutions worldwide. The study project was a genome-wide association and identified common variants in genome and associated brain volume difference. With rapid advancements in gene studies, this article was also cited in many gene and behavior-related studies.

The third-ranked article was authored by Thomas et al. and titled “Prevalence of Attention-Deficit/Hyperactivity Disorder: A Systematic Review and Meta-Analysis,” which was published in 2015 [2]. It was cited 194 times. Since over-diagnosis or under-diagnosis of ADHD is widely debated, in this paper, the author reviewed previously published papers and concluded that the prevalence rate of ADHD was 7.2%. It set a benchmark prevalence estimate for ADHD.

### Strengths and limitations

The strength of this study is that the authors clustered different variables into one picture using SNA displayed on Google Maps, which highlighted the most dominant entities in which the authors were interested. Readers can manipulate the links to better understand the association between the entities the authors are concerned about in this study. Besides, using MeSH terms to predict the number of article citations is a useful feature to identify the most dominant article type and topic category in the field of ADHD, which helps future academic pursuits in the psychiatric field. The research approach used in this study can be applied to other topics or disciplines, not just limited to the field of ADHD.

Nonetheless, there are still some limitations in this study. First, the database was exclusively extracted from PMC. The results of this study might be different if the articles were extracted from other major citation databases, such as Scopus, Web of Science, and Embase. Second, the authors used total citations as the measurement of impact as of May 29, 2020. As time goes by, the older the articles are, the more citations they may receive from citing articles.

Third, citation count does not directly reflect the quality of an article but enables a quantitative evaluation of the scientific impact of an article in a designated field. Thus, although citation statistics have been frequently criticized, the analysis of citation rates allows identification of advances in a specialty and may provide a historical perspective on its scientific progress.

Fourth, there are numerous extrinsic factors that influenced the number of article citations. Using MeSH terms to predict future citation count based on the top 100 most-cited articles might have some limitations and bias. More factors should be considered to reach a more valid prediction in the future.

## Conclusions

This study was the first to report the characteristics of the top 100 most-cited articles on ADHD using SNA on Google Maps. The results of this study not only provide a historical perspective on scientific evolution but also suggest research trends of key topics and clinical practice in the field of ADHD. This study utilized the PMC in identifying the most important articles on ADHD. The authors hope that the recent era of EBM will influence the quality of articles in ADHD research.

## Supplementary Information

**Additional file 1.**

## Data Availability

All figures presented in this study were produced by the authors. No any copy-right issue was occurred or involved in this study, including the dashboards laid on Google Maps. It is because the use of Google Maps has been permitted by Google team in advance. Additional file 1 (MP4 video) at https://youtu.be/uRyhGvBKYuc.

## Abbreviations

ADHD: Attention-deficit/hyperactivity disorder
PMC: PubMed Central^®^
SNA: Social network analysis
CC: Correlation coefficients
IF: Impact factors
